# Identification of stably expressed housekeeping miRNAs in endothelial cells and macrophages in an inflammatory setting

**DOI:** 10.1038/s41598-019-49241-7

**Published:** 2019-09-04

**Authors:** Fabian Link, Knut Krohn, Julia Schumann

**Affiliations:** 1 Clinic for Anaesthesiology and Operative Intensive Care, University Hospital Halle (Saale), Halle, Saale Germany; 2 Core Unit DNA Technologies, Medical Faculty, Leipzig University, Leipzig, Germany

**Keywords:** miRNAs, Molecular medicine

## Abstract

Reliable quantification of miRNA expression by qRT-PCR crucially depends on validated housekeepers for data normalization. Here we present thoroughly tested miRNAs eligible as references in immunological studies utilizing endothelial cells and macrophages, respectively. Endothelial cells (cell line: TIME) and macrophages (cell line: RAW264.7) were treated with various pro- and anti-inflammatory mediators (cytokines, LPS, unsaturated fatty acids) given as either single substances or in combination. Isolated RNA was screened for stably expressed miRNAs by next generation sequencing. Housekeeper candidates were thereafter validated by means of two independent quantification techniques: qRT-PCR for relative quantification and ddPCR for absolute quantification. Both methods consistently confirmed the suitability of let-7g-5p, let-7i-5p, miR-127-3p and miR-151a-5p in cytokine/fatty acid-treated TIME and miR-16-5p, miR-27b-3p, miR-103a-3p and miR-423-3p in LPS/fatty acid-treated RAW264.7, respectively as housekeeping miRNAs. With respect to abundancy and over all expression stability the miRNAs miR-151a-5p (cell line: TIME) as well as miR-27b-3p and miR-103a-3p (cell line: RAW264.7) can be particularly recommended for normalization of qRT-PCR data.

## Introduction

MicroRNAs (miRNAs) have become an object of intense research in biomedical sciences as they play an important role in the regulation of complex biological systems by repressing gene translation^1–3^. For example, miRNAs are involved in inflammatory processes and have been linked to endothelial dysfunction^4–6^ and macrophage polarization^7–9^. Further on, miRNA expression alterations have been described in oncological^10^ or various other diseases^11,12^ which makes them a promising tool in clinical diagnostics. In order to gain a detailed molecular and mechanistic understanding of miRNA functions as well as to verify the usefulness of miRNAs as innovative diagnostic biomarkers precise quantification tools are imperatively required.

A method often used for miRNA quantification is the Real-Time polymerase chain reaction (qRT-PCR)^13,14^. Modern qRT-PCR systems detect the fluorescent signal of a reporter dye after every cycle and determine the cycle in which the signal exceeds an arbitrarily set threshold^15^. This cycle number (called “Ct value”) is a measuring unit for the target abundance relative to a defined housekeeping nucleic acid which is used for normalization^16^. An absolute prerequisite to reliably compensate procedural and biological variability is the independency and stability of the chosen housekeeper^17–20^. The selection of an adequate housekeeper, therefore, is a key point in qRT-PCR analysis. However, with respect to miRNA quantification this has proven to be a difficult issue.

Housekeeping genes typically used for messenger RNA (mRNA) quantification like GAPDH and β-actin are considerably larger than miRNAs. As the amplification efficiency depends on DNA size these housekeepers are inappropriate for miRNA qRT-PCR^21^. Small nuclear and nucleolar RNAs, such as U6 or SNORD44, are more suitable with respect to size but their eligibility has been found to be limited, too. There is evidence that the expression rate of these housekeepers differs among tissue types and shows inter-individual variations^22^. In addition, these normalizers seem to be affected by the surrounding milieu, e.g. the presence of cytokines^23–25^. Another problem is that the nuclear/nucleolar RNAs differ from miRNAs in biochemical properties which could lead to normalization bias^26,27^. Housekeepers belonging to the same type of RNA, therefore, appear to be particularly appropriate for normalization. Hence, there is a need for the identification of stably expressed housekeeper miRNAs with respect to the experimental setting.

Here we present thoroughly tested miRNAs eligible as housekeepers in immunological studies utilizing endothelial cells and macrophages, respectively. The expression rate of the identified housekeeper miRNAs was proven to be virtually unaffected by several pro- and anti-inflammatory mediators (cytokines, LPS, unsaturated fatty acids) given as either single substances or in combination.

## Results

### Next generation sequencing (NGS)

To identify adequate housekeeping genes for qRT-PCR on endothelial cells (cell line: TIME) and macrophages (cell line: RAW264.7) under inflammatory conditions a NGS-based screening was carried out. In TIME treated with the cytokines IL-1β, TNF-α and IFN-γ (5 ng/ml each) 1173 different miRNAs were detected compared with 1093 miRNAs in unstimulated control cells. In RAW264.7 treated with LPS (1 µg/ml) 236 different miRNAs were detected compared with 235 miRNAs in unstimulated control cells. For those miRNAs detectable in both stimulated and unstimulated cells the coefficient of variation (CV) and NormFinder stability value over all samples was calculated to find the most stable expressed ones. Low CV and stability value indicate a stable miRNA expression. The five miRNAs with the lowest CV and stability value were elected for both cell types as potential housekeeper candidates (Table 1).

**Table 1:** Potential housekeeping miRNAs selected from screening based on NGS.

Cell line	miRNA	Coefficient of variation	Stability value
TIME	let-7g-5p	0.13	0.20
TIME	let-7i-5p	0.14	0.19
TIME	miR-106b-3p	0.14	0.16
TIME	miR-127-3p	0.14	0.20
TIME	miR-151a-5p	0.09	0.09
RAW264.7	miR-10a-5p	0.11	0.12
RAW264.7	miR-16-5p	0.09	0.12
RAW264.7	miR-27b-3p	0.10	0.11
RAW264.7	miR-103a-3p	0.10	0.12
RAW264.7	miR-423-3p	0.09	0.09

### Quantitative real-time PCR (qRT-PCR)

Eligibility of selected miRNAs as housekeepers under the influence of inflammatory mediators was validated by means of qRT-PCR. The melting curves confirmed the specificity of all primers used. An amplification efficiency between 0.90 and 1.10 was considered sufficient.

**Figure 1:**
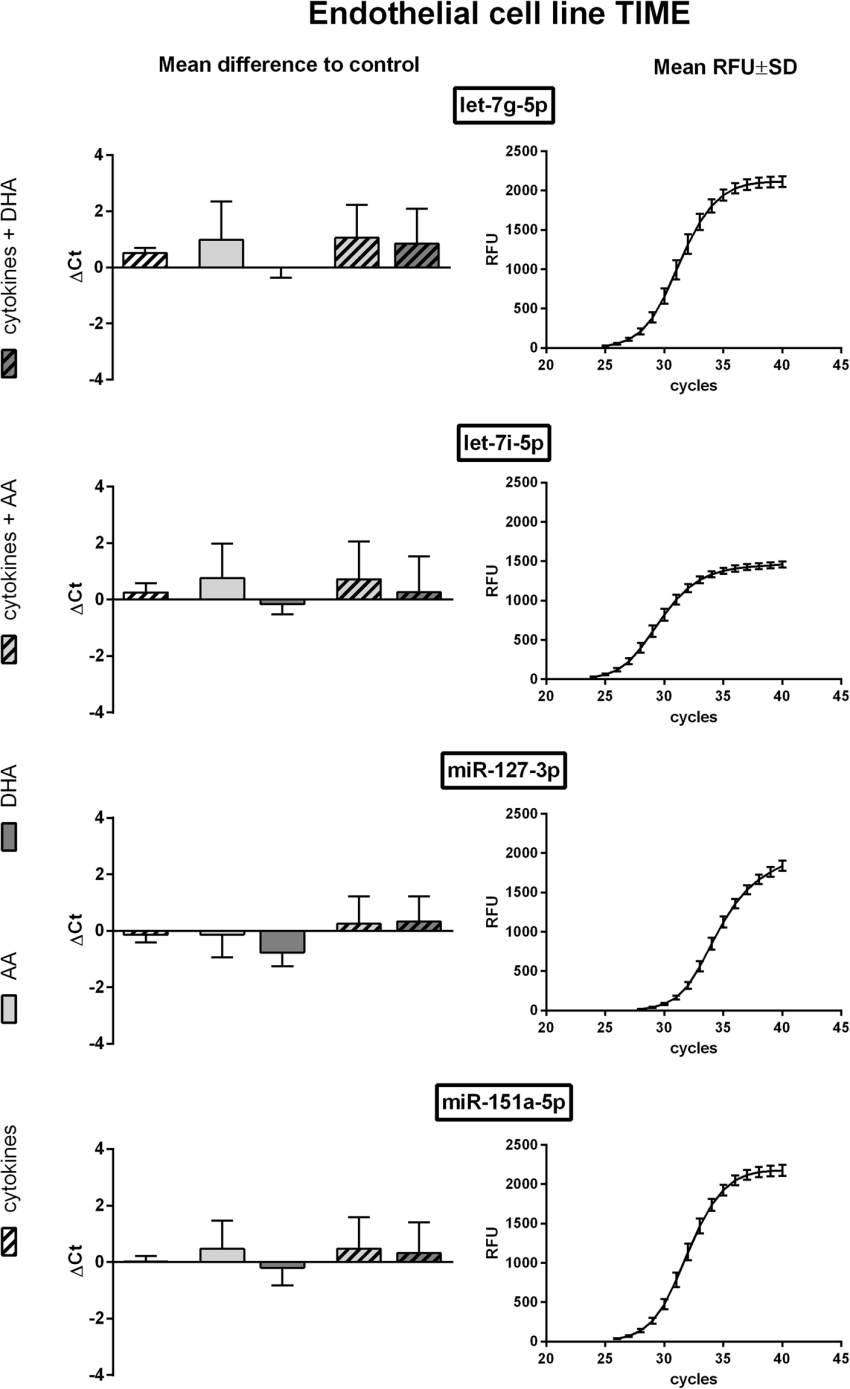
qRT-PCR data of TIME cells. TIME were stimulated with a cytokine mix consisting of IL-1β, TNF-α and IFN-γ (each in a concentration of 5 ng/ml) and/or supplemented with the unsaturated fatty acids docosahexaenoic acid (DHA, C22:6n3) or arachidonic acid (AA, C20:4n6). Quantification of miRNA expression was performed by means of the miRCURY LNA SYBR Green PCR kit and appropriate miRCURY LNA miRNA PCR assay primers on a CFX96 Touch Real Time PCR detection system (N = 4, n = 2). The fluorescence signal of all samples was measured at the end of every PCR cycle as relative fluorescent unit (RFU). miRNA expression is displayed as mean difference to unstimulated control on the basis of raw Ct values. The mean RFU values and standard deviations are plotted as a sigmoidal curve.

All of the five housekeeper candidates for TIME were detected in qRT-PCR while they differed substantially in abundance. The highest expression rate was measured for let-7i-5p with a mean Ct value of 26.52. Lowest abundancy was found for miR-106b-3p with a mean Ct value of 35.25. As expected from NGS screening data expression rates of the miRNAs let-7g-5p, let-7i-5p, miR-127-3p and miR-151a-5p were not significantly affected by cytokine stimulation (Fig. 1). Likewise, supplementation of unsaturated fatty acids or a combination of cytokine and fatty acid treatment did not alter the expression rate (Fig. 1). Of those miRNAs with a mean Ct value below 30, miR-151a-5p showed the lowest over all variation at implemented experimental conditions. Unlike the aforementioned miRNAs the miR-106b-3p displayed significant group differences between treatment groups (Supplementary Fig. S1) which render it unsuitable as a housekeeper.

**Figure 2:**
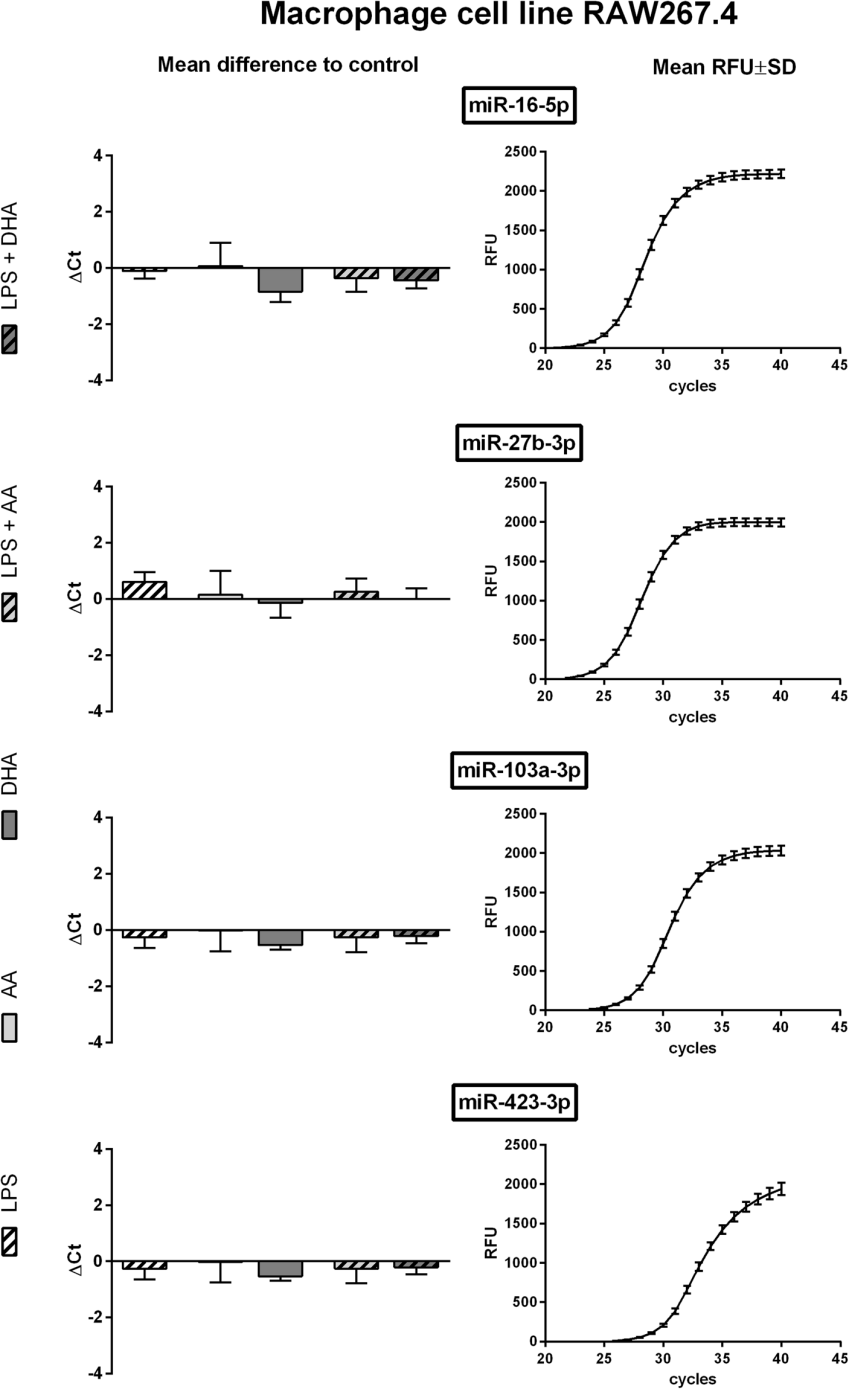
qRT-PCR data of RAW264.7 cells. RAW264.7 were stimulated with LPS (1 µg/ml) and/or supplemented with the unsaturated fatty acids docosahexaenoic acid (DHA, C22:6n3) or arachidonic acid (AA, C20:4n6). Quantification of miRNA expression was performed by means of the miRCURY LNA SYBR Green PCR kit and appropriate miRCURY LNA miRNA PCR assay primers on a CFX96 Touch Real Time PCR detection system (N = 4, n = 2). The fluorescence signal of all samples was measured at the end of every PCR cycle as relative fluorescent unit (RFU). miRNA expression is displayed as mean difference to unstimulated control on the basis of raw Ct values. The mean RFU values and standard deviations are plotted as a sigmoidal curve.

For RAW264.7 qRT-PCR-based detection was successful for each of the five housekeeping candidates. miR-27b-3p was found to be the most abundant one with a mean Ct value of 25.98. The lowest expression rate was detected for miR-10a-5p with a mean Ct value of 35.44. As displayed in Fig. 2 the miRNAs miR-16-5p, miR-27b-3p, miR-103a-3p and miR-423-3p were nearly unaltered under the influence of LPS stimulation, unsaturated fatty acid supplementation or combination treatment. Of those miRNAs with a mean Ct value below 30, miR-27b-3p and miR-103a-3p were the most invariant ones. On the contrary miR-10a-5p showed some variations in expression rates due to cytokine and/or fatty acid treatment (Supplementary Fig. S2).

### Droplet digital PCR (ddPCR)

For a deeper insight on expression changes of housekeeper candidates we additionally performed an absolute quantification of the miRNAs by means of the Droplet Digital PCR (ddPCR) technology.

**Figure 3:**
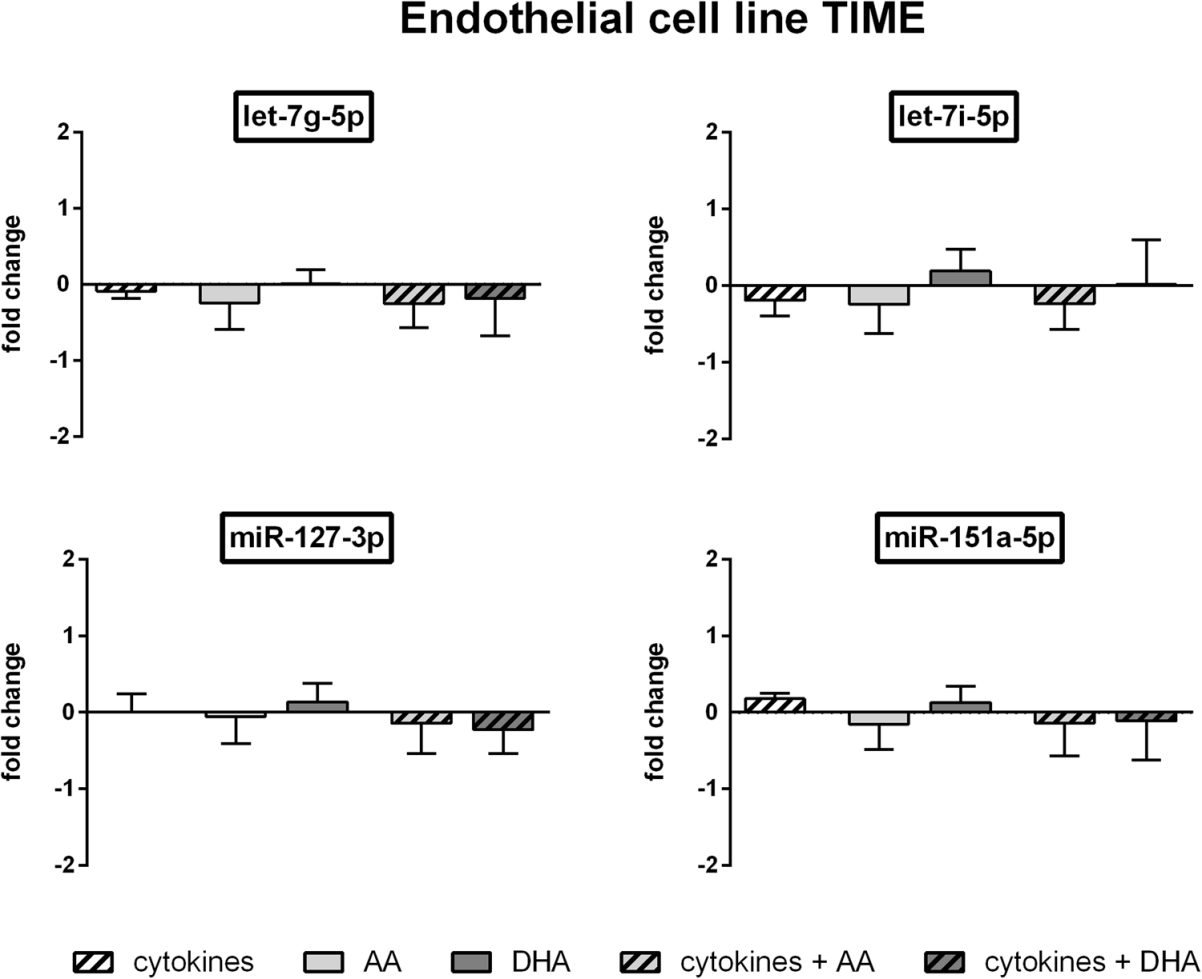
ddPCR data of TIME cells. TIME were stimulated with a cytokine mix consisting of IL-1β, TNF-α and IFN-γ (each in a concentration of 5 ng/ml) and/or supplemented with the unsaturated fatty acids docosahexaenoic acid (DHA, C22:6n3) or arachidonic acid (AA, C20:4n6). Absolute quantification of miRNA expression was performed by means of the EvaGreen Supermix and appropriate miRCURY LNA miRNA PCR assay primers on a QX200 ddPCR droplet reader system (N = 4, n = 2). miRNA expression was measured as RNA molecules per µl sample. Results are displayed as fold change to unstimulated control.

In line with the results from the qRT-PCR in TIME cells let-7i-5p showed the highest abundancy (25089 copies per ng RNA on average of all test groups) and miR-106b-3p the lowest abundancy (380 copies per ng RNA on average of all test groups) of the analysed miRNAs. The ddPCR analysis further confirmed the eligibility of the miRNAs let-7g-5p, let-7i-5p, miR-127-3p and miR-151a-5p as housekeepers under the influence of cytokines and/or unsaturated fatty acids (Fig. 3). The fold changes in miRNA expression rate induced by cytokine treatment and/or unsaturated fatty acid supplementation did not exceed 0.25 for all of these miRNAs (Fig. 3) which is also evident from the low average differences in absolute copy counts of stimulated compared to unstimulated TIME cells (Table 2). For miR-106b-3p the fold change in miRNA expression rate was found to be higher but not significantly altered compared to untreated control (Supplementary Fig. S1).

**Table 2:** miRNA copy count differences induced by inflammatory mediators (cytokines, LPS, unsaturated fatty acids).

Cell type	miRNA	Mean copy count of untreated control	Mean difference induced by LPS/cytokines and/or fatty acids
TIME	let-7g-5p	15111	2332
TIME	let-7i-5p	27198	4807
TIME	miR-127-3p	1675	189
TIME	miR-151a-5p	8965	1279
RAW264.7	miR-16-5p	105490	21978
RAW264.7	miR-27b-3p	164010	51403
RAW264.7	miR-103a-3p	19291	2841
RAW264.7	miR-423-3p	4667	545

**Figure 4:**
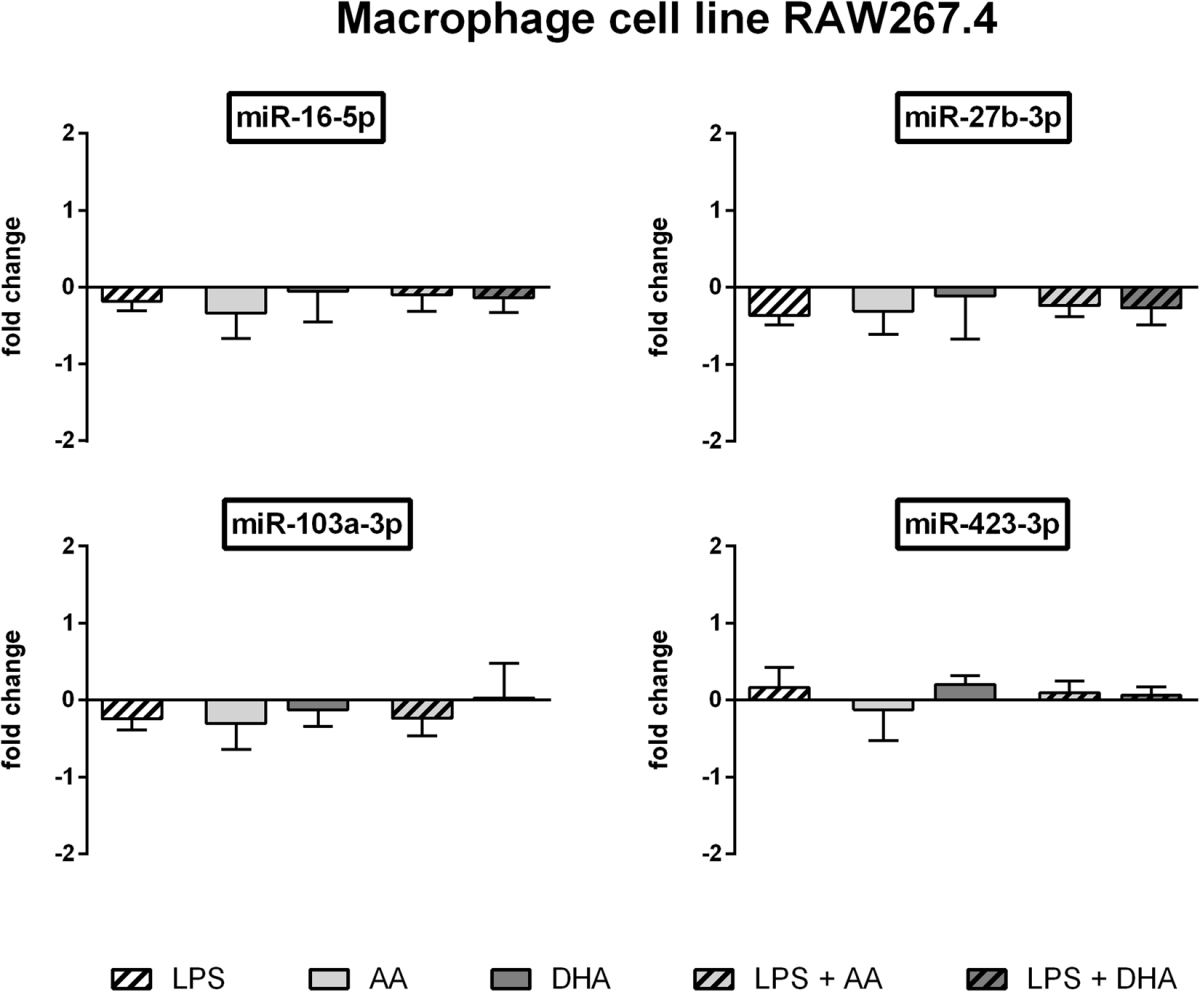
ddPCR data of RAW264.7 cells. RAW264.7 cells were stimulated with LPS (1 µg/ml) and/or supplemented with the unsaturated fatty acids docosahexaenoic acid (DHA, C22:6n3) or arachidonic acid (AA, C20:4n6). Absolute quantification of miRNA expression was performed by means of the EvaGreen Supermix and appropriate miRCURY LNA miRNA PCR assay primers on a QX200 ddPCR droplet reader system (N = 4, n = 2). miRNA expression was measured as RNA molecules per µl sample. Results are displayed as fold change to unstimulated control.

Likewise for RAW264.7 a high consistency was seen between qRT-PCR and ddPCR data. With a mean of 121174 copies per ng RNA miR-27b-3p was the most abundant of the miRNAs analysed. The lowest expression rate (1975 copies per ng RNA on average of all test groups) was found for miR-10a-5p. LPS stimulation and/or unsaturated fatty acid supplementation did not lead to significant alterations in the expression of miR-16-5p, miR-27b-3p, miR-103a-3p and miR-423-3p (Fig. 4). The induced fold change in miRNA expression rate did not exceed 0.40 for all of these miRNAs (Fig. 4). This can also be deduced from the average differences in absolute miRNA copy counts of LPS and/or fatty acid treated RAW264.7 compared to unstimulated controls (Table 2). Expression rate of miR-10a-5p was significantly affected by LPS and/or unsaturated fatty acid treatment (Supplementary Fig. S2).

## Discussion

Due to their important role in gene regulation and their potential as diagnostic markers miRNAs have become an important research area in recent years. For quantification of miRNAs qRT-PCR is a common tool providing sensitive and reliable data. However, a basic condition for gaining a sound database is the application of control measures and a proper normalization strategy. To this end different methods are available which should be used with careful consideration.

Spike-in oligonucleotides are exogenous controls added to samples in a constant concentration. They enable monitoring of cDNA synthesis efficiency and are of use for detection of inter-assay variabilities^28,29^. Normalization based on global mean expression is mainly intended for parallel measurement of multiple targets and miRNA arrays^28,29^. In experiments with a limited number of target miRNAs the usability of this method is restricted with respect to the small data record^28,29^. Housekeepers, in contrast, may even be used for normalization of single target qRT-PCR experiments. These nucleic acids are known to be stably expressed and, therefore, indicate potential intra-assay and inter-assay variabilities^28,29^. Yet, certain basic conditions have to be met in order to prevent normalization bias. The main criterion for suitability of a housekeeper is a stable expression rate with respect to the analysed cell/tissue type as well as the experimental setting. Further on, an optimal level of conformity in biochemical properties needs to be reached. For that reason housekeeper and target should be of the same RNA type.

A few reports describe the successful application of housekeeping miRNAs in certain disease models and experimental settings investigating particular cells/tissues and treatment conditions (Supplementary Table S1). It should be noted that these studies show considerable variations in the proposed reference miRNAs. This could be attributed to the fact that miRNA expression is described to not only vary among cell and tissue types but also to depend on the surrounding milieu^30–32^. It seems that there is no miRNA that is eligible as universal housekeeper. Thus, there is a need for defining adequate housekeeping miRNAs with respect to the intended model system. The present study was undertaken in order to establish valid housekeepers for miRNA studies on endothelial cells or macrophages in an inflammatory setting.

Three different techniques were used in a systematically manner. First, NGS was applied to screen all expressed miRNAs (1173 miRNAs for endothelial cell line TIME, 236 miRNAs for macrophage cell line RAW264.7) for their potential eligibility as housekeepers. For both cell lines the five most invariant miRNAs were selected for further validation. Candidates identified were let-7g-5p, let-7i-5p, miR-106b-3p, miR-127-3p and miR-151a-5p for TIME as well as miR-10a-5p, miR-16-5p, miR-27b-3p, miR-103a-3p and miR-423-3p for RAW264.7.

In the next step those miRNAs were analysed using two independent quantification methods: relative quantification by means of qRT-PCR and absolute quantification by means of ddPCR. Both techniques consistently confirmed the suitability of the above mentioned miRNAs as housekeepers in the presence of cytokines/LPS and/or unsaturated fatty acids except for miR-106b-3p (TIME) and miR-10a-5p (RAW264.7). In particular, miR-151a-5p in TIME as well as miR-27b-3p and miR-103a-3p in RAW264.7 can be highly recommended as housekeeping miRNAs with respect to abundancy and over all expression stability.

Previous studies propose the use of the geometric mean of multiple housekeepers as an elegant option to minimize normalization bias^20,33,34^. The validation of four housekeeping miRNAs for both cell types analysed, therefore, represents an opportunity to further optimize qRT-PCR-based quantification.

Remarkably, the majority of the miRNAs identified in our study have already been proposed as housekeepers in the literature (Supplementary Table S1). Whether the identified reference miRNAs might be applied to further cell/tissue types and experimental conditions carefully needs to be validated before being used in any deviant settings. Yet, it is probable that let-7g-5p, let-7i-5p and miR-151a-5p are generally suitable as housekeeping miRNAs in immunological studies investigating vessel cells of mammalian origin. Complementary investigations of our laboratory provides evidence that these miRNAs are stably expressed not only in the endothelial cell line TIME but also in primary murine endothelial cells (mEC) and vascular smooth muscle cells (mVSMC) in either presence or absence of cytokine stimulation (Supplementary Fig. S3).

In order to check the plausibility of the results, the biological function of the proposed reference miRNAs was assessed based on in silico prediction of genomic binding sites from mirWalk^35^. For GO enrichment focussed on the ontology domain “biological process” G:profiler^36^ was used and redundant GO terms were removed by subset analysis by means of REVIGO^37^. Altogether 546 GO term subsets in the biological process domain could be identified. As expected, interrelations of each miRNA with biological processes are diverse and complex. Nevertheless certain functionalities are frequently present. For example, all proposed miRNAs are involved in processes at the neuronal synapse like “synapse organization” (GO:0050808) and “learning and memory” (GO:0007611). Another, frequently occurring pattern is the functional connection to formation of the urogenital tract (GO:0001655) and reproductive system (GO:0061458). A link to inflammatory processes is only found for miR-423-3p as terms like “immune effector process” (GO:0002252) or “leucocyte differentiation” (GO:0002521) are assigned to this miRNA. In this respect, the in silico data are consistent with the results of our study, as the putative and validated functional relations, with the exception of miR-423-3p, do not suggest any change due to an inflammatory milieu.

Over and above the presented data underlines the suitability of ddPCR for a housekeeper-independent absolute quantification of miRNAs. The method has shown to deliver highly reliable and comparable results without necessity of any reference for normalization. In spite of the extra effort and cost this technique might be of benefit in case of unsatisfactory housekeepers.

## Materials and Methods

### Cell lines and culture media

If not stated otherwise, materials were provided by Sigma-Aldrich (Taufkirchen, Germany). Commercially available cell lines (LGC Standards, Wesel, Germany) were used: the human telomerase-immortalized microvascular endothelial cell line TIME (ATCC number: CRL-4025) as well as the murine macrophage cell line RAW264.7 (ATCC number: TIB-71). TIME were cultured in basal microvascular endothelial cell growth medium (Provitro, Berlin, Germany) enriched with 5 ng/ml VEGF, 5 ng/ml EGF, 5 ng/ml FGF, 15 ng/ml IGF-1, 10 mM L-glutamine, 0.75 U/ml heparin sulphate, 1 µg/ml hydrocortisone hemisuccinate, 50 µg/ml ascorbic acid (all Provitro, Berlin, Germany), 5% v/v FCS, and 12.5 µg/ml blasticidin (Invitrogen/Thermo Fischer Scientific, Dreieich, Germany). Cell culture medium used for RAW264.7 was RPMI 1640 (PAN-Biotech, Aidenbach, Germany) enriched with 5% v/v fetal calf serum (FCS).

### Fatty acid supplementation and stimulation of cells

For cellular fatty acid enrichment TIME as well as RAW264.7 were incubated in cell culture medium supplemented with either docosahexaenoic acid (DHA, C22:6n3) or arachidonic acid (AA, C20:4n6) (both Biotrend, Köln, Germany) in a final concentration of 15 µM for 72 hours. For stimulation TIME were treated with a cytokine mix consisting of IL-1β, TNF-α and IFN-γ, each in a concentration of 5 ng/ml (all PeproTech, Hamburg, Germany) and RAW264.7 were treated with 1 µg/ml LPS (from E. coli serotype 0111:B4). Stimulation was performed in the last 24 hours of fatty acid supplementation.

### RNA isolation

Total RNA isolation was performed using TriFast FL (VWR/Peqlab, Erlangen, Germany) following the manufacturer’s standard protocol. RNA concentration and quality was determined by means of a NanoDrop spectrophotometer (Thermo Fischer Scientific, Dreieich, Germany). An absorbance quotient A260/280 > 1.8 was considered appropriate for following procedures. Complementary DNA (cDNA) was synthesized by means of the miRCURY LNA RT Kit (QIAGEN, Hilden, Germany) according to standard protocol. Synthesized cDNA was used for both qRT-PCR and ddPCR miRNA expression quantification.

### Deep sequencing and analysis of deep sequencing data

miRNA expression analysis was performed using high-throughput sequencing by means of an Illumina HiScanSQ (Illumina Inc., San Diego, USA) in the Core Unit DNA at the Medical Faculty, Leipzig University. In every test group three biological replicates were analysed. Samples were processed using the TruSeq Small RNA Prep kit v2 (Illumina Inc., San Diego, USA) following the manufacturer’s standard protocol. Size restriction (140–165 bp), purification and quantification of barcoded libraries were performed using the Library quantification kit - Illumina/Universal (KAPA Biosystems, Woburn, USA). For cluster generation up to 10 libraries per lane were factored using an Illumina cBot. 50 bp sequencing was performed using an Illumina HighScanSQ sequencer based on version 3 chemistry and flowcell following the manufacturer’s standard protocol.

For deep sequencing data analysis the adapter sequences were removed from raw sequences by means of Cutadapt software, version 1.9.1^38^. To verify that small RNAs other than miRNAs are filtered out from the data only sequences 15–27 bases long were analysed. These reads were aligned to human (GRCh38: NCBI_Assembly:GCA_000001405.15) and murine (GRCm38): NCBI_Assembly:GCA_000001635.2) genome as well as mature sequences of miRBase v21 using the bowtie2 aligner^39^. An error ratio of 1 nt per mature miRNA sequence was accepted. For data compression to bam format Samtools^40^ were used. Mapped reads count was determined using the R/Bioconductor programming environment^41^ by application of the ShortRead library^42^. Sequence analysis after mapping against mature sequences of miRBase v21 is not affected by counts for other small RNAs (e.g rRNA, tRNA fragments) because these sequences are not present in the reference sequences. For mapping against the whole genome reference rRNA or tRNA fragments are usually below 1–2% in the 15–27 bp fraction. Reads that map to their respective loci were removed before analysis using genome coordinates. Normalization of data was performed by independent application of the DESeq 2 and the TMM (EdgeR) algorithm^43^.

Stability of miRNA expression was assessed based on coefficient of variation (CV), which is defined as the ratio of standard deviation to the mean. In addition the bioinformatic VBA tool NormFinder v.0.953 for Microsoft Excel was used to confirm expression stability considering the intra- and intergroup variation^44^.

### Quantitative real-time PCR (qRT-PCR)

Relative expression of miRNAs was analysed by means of the miRCURY LNA SYBR Green PCR kit and appropriate miRCURY LNA miRNA PCR assay primers (both QIAGEN, Hilden, Germany) following the manufacturer’s instructions. Thermal cycling was performed on a CFX96 Touch Real Time PCR detection system (BioRad, Munich, Germany). The cycling conditions comprised initial heat activation at 95 °C for 2 minutes, 40 cycles at 95 °C for 10 seconds and 56 °C for 60 seconds followed by a melt curve analysis. qRT-PCR reactions were performed in duplicates of four biological replicates in every test group.

### Droplet digital PCR (ddPCR)

miRNA copy counts were determined by means of the Droplet Digital PCR technology (BioRad, Munich, Germany) following the manufacturer’s standard protocols. Appropriate miRCURY LNA miRNA PCR assay primers (QIAGEN, Hilden, Germany) and ddPCR EvaGreen Supermix (Bio-Rad, Munich, Germany) were used. Measurement of the nucleic acid copy count per µl sample was performed on a QX200 ddPCR Droplet Reader (Bio-Rad, Munich, Germany). Data output was converted into nucleic acid copy count per ng RNA. ddPCR reaction was performed in duplicates of four biological replicates in every test group.

### Statistical analysis

Data are presented as means ± standard deviation (SD). To identify statistical differences between means one-way analysis of variance and multiple comparisons with Tukey correction was used. Statistical analysis was performed by means of the GraphPad Prism 6 statistics software (GraphPad Software, La Jolla, USA). A p value < 0.05 was assumed as an indicator of significant differences.

## Supplementary information

Supplementary Information

## Data Availability

Deep sequencing datasets generated and analysed during the current study are available in the Gene Expression Omnibus (GEO) repository, accession number GSE132361.

## References

[1] Bartoszewski R, Sikorski AF (2018). Editorial focus: entering into the non-coding RNA era. Cell Mol Biol Lett.

[2] Gurtan AM, Sharp PA (2013). The role of miRNAs in regulating gene expression networks. J Mol Biol.

[3] Moreno-Moya JM, Vilella F, Simon C (2014). MicroRNA: key gene expression regulators. Fertil Steril.

[4] Harris TA, Yamakuchi M, Ferlito M, Mendell JT, Lowenstein CJ (2008). MicroRNA-126 regulates endothelial expression of vascular cell adhesion molecule 1. Proc Natl Acad Sci USA.

[5] Loyer X (2014). Inhibition of microRNA-92a prevents endothelial dysfunction and atherosclerosis in mice. Circ Res.

[6] Nemecz M, Alexandru N, Tanko G, Georgescu A (2016). Role of MicroRNA in Endothelial Dysfunction and Hypertension. Curr Hypertens Rep.

[7] Liu G, Abraham E (2013). MicroRNAs in immune response and macrophage polarization. Arterioscler Thromb Vasc Biol.

[8] Wu XQ (2016). Emerging role of microRNAs in regulating macrophage activation and polarization in immune response and inflammation. Immunology.

[9] Essandoh K, Li Y, Huo J, Fan GC (2016). MiRNA-Mediated Macrophage Polarization and its Potential Role in the Regulation of Inflammatory Response. Shock.

[10] Wang H, Peng R, Wang J, Qin Z, Xue L (2018). Circulating microRNAs as potential cancer biomarkers: the advantage and disadvantage. Clin Epigenetics.

[11] Wang J, Chen J, Sen S (2016). MicroRNA as Biomarkers and Diagnostics. J Cell Physiol.

[12] Kumar P (2013). Circulating miRNA biomarkers for Alzheimer’s disease. PLoS One.

[13] Chen, C., Tan, R., Wong, L., Fekete, R. & Halsey, J. In *PCR Protocols* (ed Daniel J. P.) 113–134 (Humana Press, 2011).

[14] Kong W, Zhao JJ, He L, Cheng JQ (2009). Strategies for profiling microRNA expression. J Cell Physiol.

[15] Ginzinger DG (2002). Gene quantification using real-time quantitative PCR: an emerging technology hits the mainstream. Exp Hematol.

[16] Livak KJ, Schmittgen TD (2001). Analysis of relative gene expression data using real-time quantitative PCR and the 2(-Delta Delta C(T)) Method. Methods.

[17] Schmittgen TD, Livak KJ (2008). Analyzing real-time PCR data by the comparative C(T) method. Nat Protoc.

[18] Rice J, Roberts H, Rai SN, Galandiuk S (2015). Housekeeping genes for studies of plasma microRNA: A need for more precise standardization. Surgery.

[19] Morata-Tarifa C (2017). Validation of suitable normalizers for miR expression patterns analysis covering tumour heterogeneity. Sci Rep.

[20] Schwarzenbach H, da Silva AM, Calin G, Pantel K (2015). Data Normalization Strategies for MicroRNA Quantification. Clin Chem.

[21] Mase M (2017). Selection of reference genes is critical for miRNA expression analysis in human cardiac tissue. A focus on atrial fibrillation. Sci Rep.

[22] Xiang M (2014). U6 is not a suitable endogenous control for the quantification of circulating microRNAs. Biochem Biophys Res Commun.

[23] Benz F (2013). U6 is unsuitable for normalization of serum miRNA levels in patients with sepsis or liver fibrosis. Exp Mol Med.

[24] D’Haene B, Mestdagh P, Hellemans J, Vandesompele J (2012). miRNA expression profiling: from reference genes to global mean normalization. Methods Mol Biol.

[25] Reid G, Kirschner MB, van Zandwijk N (2011). Circulating microRNAs: Association with disease and potential use as biomarkers. Crit Rev Oncol Hematol.

[26] Gee HE (2011). The small-nucleolar RNAs commonly used for microRNA normalisation correlate with tumour pathology and prognosis. Br J Cancer.

[27] Chugh P, Dittmer DP (2012). Potential pitfalls in microRNA profiling. Wiley Interdiscip Rev RNA.

[28] Bustin SA (2009). The MIQE guidelines: minimum information for publication of quantitative real-time PCR experiments. Clin Chem.

[29] Nolan, T., Huggett, J. & Sanchez, E. *Good practice guide for the application of quantitative PCR (qPCR)* (2013).

[30] Benes V (2015). Identification of cytokine-induced modulation of microRNA expression and secretion as measured by a novel microRNA specific qPCR assay. Sci Rep.

[31] Das MK, Andreassen R, Haugen TB, Furu K (2016). Identification of Endogenous Controls for Use in miRNA Quantification in Human Cancer Cell Lines. Cancer Genomics Proteomics.

[32] Ludwig N (2016). Distribution of miRNA expression across human tissues. Nucleic Acids Res.

[33] Vandesompele J (2002). Accurate normalization of real-time quantitative RT-PCR data by geometric averaging of multiple internal control genes. Genome Biol.

[34] Marabita F (2016). Normalization of circulating microRNA expression data obtained by quantitative real-time RT-PCR. Brief Bioinform.

[35] Sticht C, Torre DL, Parveen C, Gretz A (2018). N. miRWalk: An online resource for prediction of microRNA binding sites. PLoS One.

[36] Raudvere U (2019). g:Profiler: a web server for functional enrichment analysis and conversions of gene lists (2019 update). Nucleic Acids Res.

[37] Supek F, Bosnjak M, Skunca N, Smuc T (2011). REVIGO summarizes and visualizes long lists of gene ontology terms. PLoS One.

[38] Martin, M. *CUTADAPT removes adapter sequences from high-throughput sequencing reads*. Vol. 17 (2011).

[39] Langmead B, Trapnell C, Pop M, Salzberg SL (2009). Ultrafast and memory-efficient alignment of short DNA sequences to the human genome. Genome Biol.

[40] Li H (2009). The Sequence Alignment/Map format and SAMtools. Bioinformatics.

[41] Gentleman RC (2004). Bioconductor: open software development for computational biology and bioinformatics. Genome Biol.

[42] Morgan M (2009). ShortRead: a bioconductor package for input, quality assessment and exploration of high-throughput sequence data. Bioinformatics.

[43] Stokowy T (2014). Analysis options for high-throughput sequencing in miRNA expression profiling. BMC Res Notes.

[44] Andersen CL, Jensen JL, Orntoft TF (2004). Normalization of real-time quantitative reverse transcription-PCR data: a model-based variance estimation approach to identify genes suited for normalization, applied to bladder and colon cancer data sets. Cancer Res.

