# Author Correction: Identification of stably expressed housekeeping miRNAs in endothelial cells and macrophages in an inflammatory setting

**DOI:** 10.1038/s41598-019-51008-z

**Published:** 2019-10-03

**Authors:** Fabian Link, Knut Krohn, Julia Schumann

**Affiliations:** 1 Clinic for Anaesthesiology and Operative Intensive Care, University Hospital Halle (Saale), Halle, Saale Germany; 2 Core Unit DNA Technologies, Medical Faculty, Leipzig University, Leipzig, Germany

Correction to: *Scientific Reports* 10.1038/s41598-019-49241-7, published online 04 September 2019

The Acknowledgements section in this Article is incomplete.

“The authors wish to thank Claudia Rößler, Anja Leimert and Melanie Cieselski for their great assistance in cell culture and RNA isolation. Special thanks to the Hans Böckler Foundation that supported this work by a scholarship (Grant number 394216).”

Should Read:

“The authors wish to thank Claudia Rößler, Anja Leimert and Melanie Cieselski for their great assistance in cell culture and RNA isolation. Special thanks to the Hans Böckler Foundation that supported this work by a scholarship (Grant number 394216). We acknowledge the financial support within the funding program Open Access Publishing by the German Research Foundation (DFG)”.

