# Supplementary material for: Identification of stably expressed housekeeping miRNAs in endothelial cells and macrophages in an inflammatory setting
Source: Sci Rep. 2019 Sep 4;9:12786. doi: 10.1038/s41598-019-49241-7 (PMC6726651; doi:10.1038/s41598-019-49241-7)
Supplement: Supplementary file 1 — Supplementary Information [file 41598_2019_49241_MOESM1_ESM.pdf]

Article

# Identification of stably expressed housekeeping miRNAs in endothelial cells and macrophages in an inflammatory setting

**Fabian Link, Knut Krohn and Julia Schumann**

# miR-106b-3p in endothelial cell line TIME

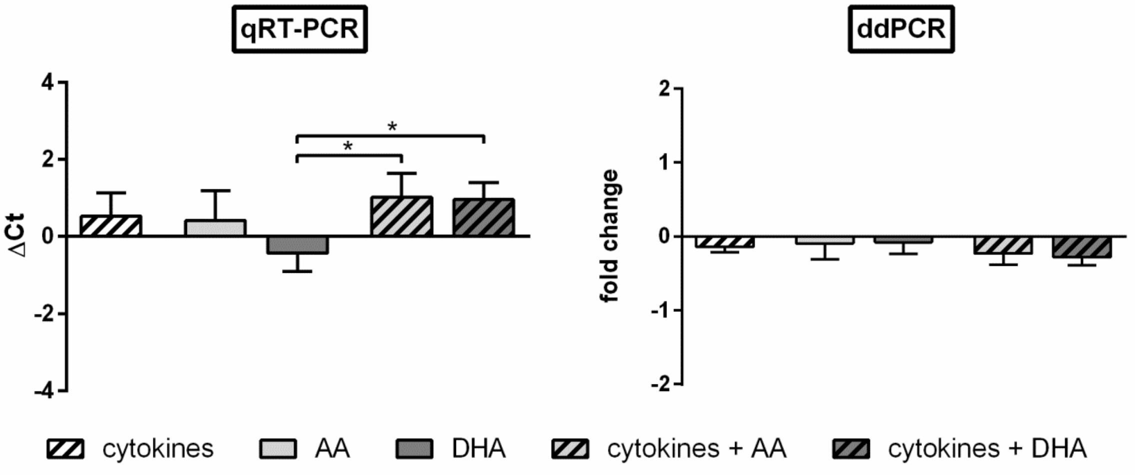


**Figure S1: Expression alterations of miR-106b-3p in endothelial cell line TIME.**
TIME were stimulated with a cytokine mix consisting of IL-1$\beta$, TNF-$\alpha$, and IFN-$\gamma$ (each in a concentration of 5 ng/ml) and/or supplemented with the unsaturated fatty acids docosahexaenoic acid (DHA, C22:6n3) or arachidonic acid (AA, C20:4n6). Relative quantification of miRNA expression (qRT-PCR) was performed by means of the miRCURY LNA SYBR Green PCR kit and appropriate miRCURY LNA miRNA PCR assay primers on a CFX96 Touch Real Time PCR detection system (N = 4, n = 2). miRNA expression is displayed as mean difference to unstimulated control on the basis of raw Ct values. Absolute quantification of miRNA expression(ddPCR) was performed by means of the EvaGreen Supermix and appropriate miRCURY LNA miRNA PCR assay primers on a QX200 ddPCR droplet reader system (N = 4, n = 2). miRNA expression was measured as RNA molecules per µl sample. Results are displayed as fold change to unstimulated control.
* $p < 0.05$; ** $p < 0.01$; *** $p < 0.001$

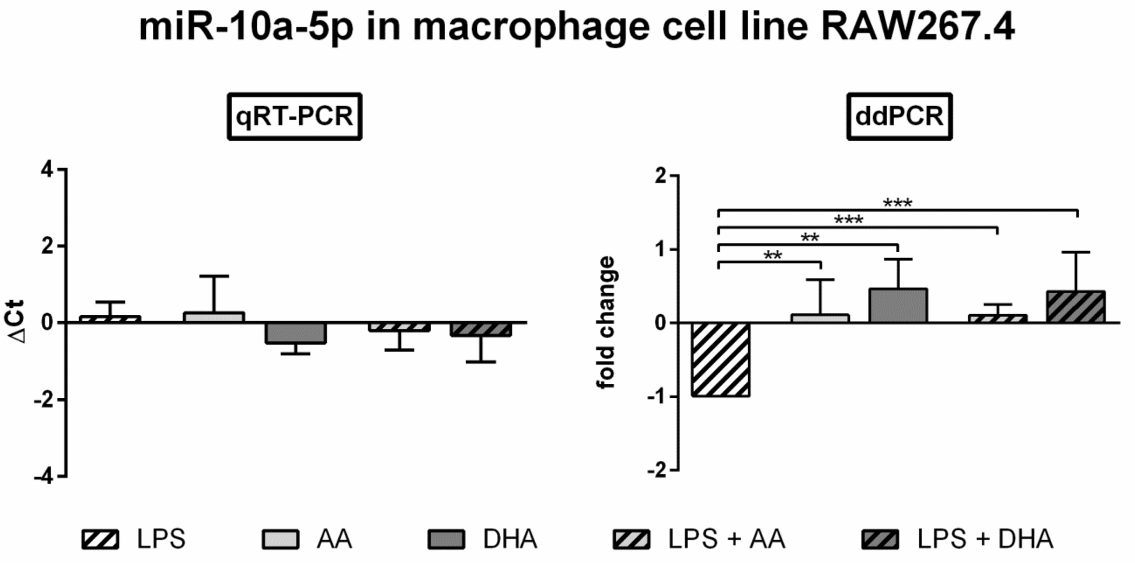


**Figure S2: Expression alterations of miR-10a-5p in macrophage cell line RAW264.7.**
RAW264.7 were stimulated with LPS (1 µg/ml) and/or supplemented with the unsaturated fatty acids docosahexaenoic acid (DHA, C22:6n3) or arachidonic acid (AA, C20:4n6). Relative quantification of gene expression (qRT-PCR) was performed by means of the miRCURY LNA SYBR Green PCR kit and appropriate miRCURY LNA miRNA PCR assay primers on a CFX96 Touch Real Time PCR detection system (N = 4, n = 2). miRNA expression is displayed as mean difference to unstimulated control on the basis of raw Ct values. Absolute quantification of miRNA expression (ddPCR) was performed by means of the EvaGreen Supermix and appropriate miRCURY LNA miRNA PCR assay primers on a QX200 ddPCR droplet reader system (N = 4, n = 2). miRNA expression was measured as RNA molecules per µl sample. Results are displayed as fold change to unstimulated control.
* $p < 0.05$; ** $p < 0.01$; *** $p < 0.001$

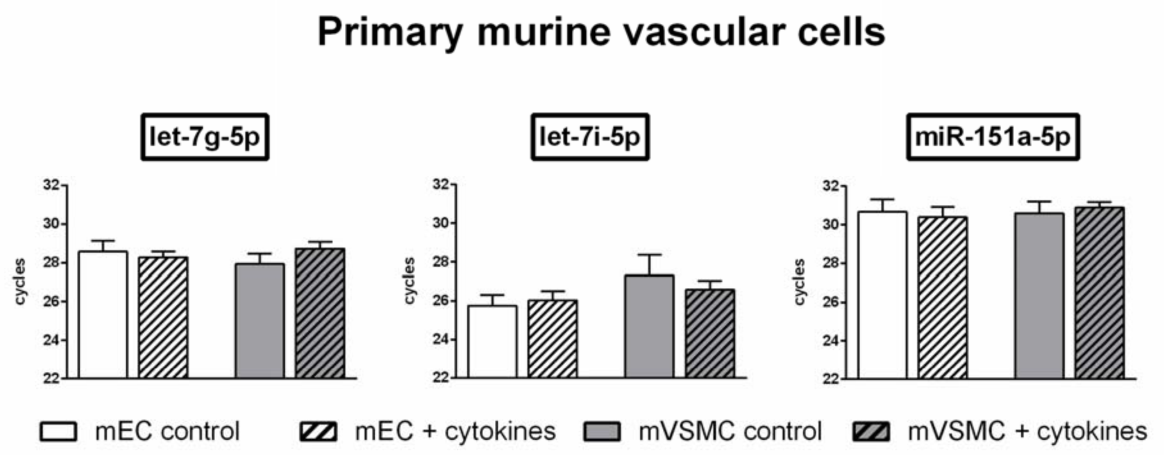


**Figure S3: Expression rates of let-7g-5p, let-7i-5p, and miR-151a-5p in primary murine vascular cells.** Murine endothelial cells (mEC) were isolated from murine mesenteries and vascular smooth muscle cells (mVSMC) from lungs of C57BL/6 mice. Cells were stimulated with a cytokine mix consisting of IL-1$\beta$, TNF-$\alpha$, and IFN-$\gamma$ (each in a concentration of 5 ng/ml). Relative quantification of miRNA expression was performed by means of the miRCURY LNA SYBR Green PCR kit and appropriate miRCURY LNA miRNA PCR assay primers on a CFX96 Touch Real Time PCR detection system (N = 4, n = 2). miRNA expression is displayed as mean difference to unstimulated control on the basis of raw Ct values.

**Table S1:** Summary of miRNAs discussed as data normalizers in different human studies

| Condition | miRNA | Source | Reference |
|---|---|---|---|
| Bladder cancer | miR-16-5p, miR-193a-5p | Serum | doi:10.3892/mmr.2015.3428 |
| | miR-101, miR-125a-5p, miR-148b, miR-151-5p, miR-181b, miR-874 | Tissue | doi:10.1371/journal.pone.0039309 |
| Breast cancer | miR-16, miR-425 | Blood | doi:10.1371/journal.pone.0083718 |
| | let-7a, miR-16 | Tissue | doi:10.1186/1471-2199-9-76 |
| | miR-16-5p, miR-29a-3p | Tissue | doi:10.3390/ijms17020156 |
| Cervical cancer | miR-23a, miR-191 | Tissue | doi:0.3858/emm.2011.43.6.039 |
| | miR-151-5p, miR-152-3p, miR-423-3p | Tissue | doi:10.1016/j.tranon.2018.12.010 |
| Chronic kidney disease | miR-16 | Urinary exosomes | doi:10.1371/journal.pone.0183435 |
| Colorectal cancer | miR-191-5p | Serum | doi:10.1371/journal.pone.0083025 |
| | miR-25-3p, miR-93-5p, miR-106b-5p | Serum | doi:10.1038/srep35611 |
| | let-7g, miR-27a, miR-193a-5p | Tissue | doi:10.1371/journal.pone.0150593 |
| | miR-16, miR-345 | Tissue | doi:10.1186/1471-2407-10-173 |
| | miR-345, miR-520d, miR-1228 | Tissue, plasma, exosomes | doi:10.1038/s41598-017-08784-3 |
| Diffuse large B-cell lymphoma | miR-24 | Tissue | doi:10.1136/jclinpath-2012-201021 |
| Dihydrotestosterone treatment | miR-16, miR-1228-3p | Prostate cells lines RWPE-1, LNCaP, 22RV1, DU145, PC-3 | doi:10.1038/s41598-018-19458-z |
| Esophageal cancer | miR-28-5p, miR-34a-5p, miR-186-5p | Tissue | doi:10.1007/s12032-016-0860-7 |
| Gastric cancer | miR-16, miR-93 | Serum | doi:10.1007/s10620-011-1981-7 |
| | miR-101-3p, miR-140-3p | Tissue | doi: 10.1016/j.gene.2017.04.016 |
| Heart failure / hypertension | let-7i, miR-16 | Serum | doi:10.21037/jtd.2018.04.128 |
| Hepatitis B | miR-22*, miR-26a, miR-221 | Serum | doi:10.1007/s12033-011-9414-6 |
| | miR-24-3p, miR-151a-5p, miR-425-5p | Cell line HepG2 | doi:10.1186/s13104-016-1848-2 |
| Kidney cancer | miR-28, miR-103, miR-106a | Tissue | doi:10.1016/j.ab.2011.06.009 |
| Low/high estrogen levels | miR-188-5p, miR-222-3p | Plasma | doi:10.1186/s13104-017-2636-3 |
| Lung cancer | miRNA-192 | Pleural effusion | doi:10.3892/ol.2014.2404 |
| Major depressive disorder | miR-93-5p, miR-101-3p | Plasma | doi:10.1016/j.jad.2013.12.035 |
| Neuronal differentiation | miR-26b, miR-103, miR-106b | Cell lines SK-N-BE(2)C, neuro-2a, NG108-15, PC12; ENStem-A neural progenitor cells | doi:10.1016/j.neuroscience.2011.10.024 |
| Ovarian cancer | miR-191-5p | Tissue | doi:10.1111/jcmm.12927 |
| Prostate cancer | miR-191 | Urine sediments | doi: 10.1155/2015/973597 |
| | miR-130b | Tissue | doi:10.3858/emm.2010.42.11.076 |
| Stable coronary artery disease | miR-4516, miR-6090 | Plasma | doi:10.5603/CJ.2016.0109 |
| Thyroid cancer | miR-99a-5p, miR-151a-3p, miR-197-3p, miR-214-3p | Aspiration smears | doi:10.3892/or.2016.5113 |
| Training status | miR-320d | Plasma | doi:10.1038/s41598-019-38505-x |
| Vulvar cancer | miR-93-5p, miR-425-5p | Plasma | doi:10.1177/1010428317717140 |
